# Cardiorespiratory fitness and the association with galectin-1 in middle-aged individuals

**DOI:** 10.1371/journal.pone.0301412

**Published:** 2024-04-05

**Authors:** Daniel Arvidsson, Vagner Ramon Rodrigues Silva, Örjan Ekblom, Elin Ekblom-Bak, Emanuel Fryk, Per-Anders Jansson, Mats Börjesson

**Affiliations:** 1 Center for Health and Performance, Department of Food and Nutrition, and Sport Science, Faculty of Education, University of Gothenburg, Gothenburg, Sweden; 2 Wallenberg Laboratory, Department of Molecular and Clinical Medicine, Institute of Medicine, Sahlgrenska Academy, University of Gothenburg, Gothenburg, Sweden; 3 Department of Physical Activity and Health, Swedish School of Sport and Health Sciences, Stockholm, Sweden; 4 Center for Lifestyle Intervention, Department of Molecular and Clinical Medicine, Institute of Medicine, Sahlgrenska Academy, University of Gothenburg, Gothenburg, Sweden; 5 Sahlgrenska University Hospital, Region Västra Götaland, Gothenburg, Sweden; University of Michigan Medical School, UNITED STATES

## Abstract

Galectin-1 plays a functional role in human metabolism and the levels are altered in obesity and type 2 diabetes (T2D). This study investigates the association of cardiorespiratory fitness (CRF) with galectin-1 and the interconnection with body fatness. Cross-sectional data from the Swedish CArdioPulmonary bioImage Study (SCAPIS) pilot was analyzed, including a sample of 774 middle-aged individuals. A submaximal cycle ergometer test was used to estimate CRF as an indirect measure of the physical activity (PA) level. Serum-galectin-1 concentration was determined from venous blood collected after an overnight fast. Body mass index (BMI) was used as an indirect measure of body fatness. CRF was significantly associated with galectin-1, when controlled for age and sex (regression coefficient (regr coeff) = -0.29, p<0.001). The strength of the association was attenuated when BMI was added to the regression model (regr coeff = -0.09, p = 0.07), while the association between BMI and galectin-1 remained strong (regr coeff = 0.40, p<0.001). CRF was associated with BMI (regr coeff = -0.50, p<0.001). The indirect association between CRF and galectin-1 through BMI (-0.50 x 0.40) contributed to 69% of total association (mediation analysis). In group comparisons, individuals with low CRF-high BMI had the highest mean galectin-1 level (25 ng/ml), while individuals with high CRF-low BMI had the lowest level (21 ng/ml). Intermediate levels of galectin-1 were found in the low CRF-low BMI and high CRF-high BMI groups (both 22 ng/ml). The galectin-1 level in the low CRF-high BMI group was significantly different from the other three groups (P<0.001). In conclusion, galectin-1 is associated with CRF as an indirect measure of the PA level through interconnection with body fatness. The size of the association is of clinical relevance.

## Introduction

Galectins are a family of carbohydrate-binding proteins highly conserved between species. There are 15 galectin isoforms and 12 of them are expressed in mammals [1]. Galectin-1 is expressed in most tissues in the body and presents a functional role in tissue reorganization, metabolism, and inflammation [2–4]. In recent years, animal and human studies highlighted galectin-1 in several diseases, including obesity, type 2 diabetes (T2D), cancer, cardiovascular and muscle diseases [3, 5, 6]. Of note, adipose tissue interstitial levels of galectin-1 are increased in patients with T2D, and high levels of serum galectin-1 are associated with an increased risk of developing T2D in longitudinal studies [7, 8]. Serum galectin-1 also increases with BMI in both children and adults, and animal studies show a function of galectin-1 in adipose tissue metabolism [9–11]. Serum galectin-1 correlates with plasma triglycerides and serum insulin, suggesting a common mechanism in the development of T2D through pathways regulating metabolism and energy balance [9].

A healthy lifestyle with regular physical activity (PA) is associated with a reduction in cardiovascular (CVD) risk factors, including insulin resistance and T2D, hypertension and high cholesterol [12]. Regular PA is also associated with reduced CVD morbidity and mortality [13]. Physical activity is typically assessed by self-report methods, which are prone to overestimation [14]. More objective measures of PA, such as accelerometry, have therefore been introduced. However, accelerometry also has limitations, and the typically short duration of data collection, up to 7 days, reduces reliability. Maximal oxygen consumption (VO_2_max) as a measure of cardiorespiratory fitness (CRF) provides a more stable indicator of habitual PA [15]. In fact, the American Heart Association (AHA) has stated that CRF is perhaps the most important risk factor for CVD, being at least as important as classical risk factors [16]. However, the mechanisms by which PA/CRF affect CVD are still not fully elucidated.

While low PA is also associated with the development of T2D, cancer, and regulation of human energy homeostasis [17, 18], there are no studies on the association between galectin-1 and PA, although a recent study found that galectin-3 is associated with VO_2_peak in men [19]. However, studies suggest a role for galectin-1 in muscle function independent of metabolism. Galectin-1 is altered in muscle disease, and is increased in sarcopenia, and histochemical studies colocalize galectin-1 with leukocytes, indicating that inflammation could be an underlying mediator [20]. In muscle dystrophy, galectin-1 appears to have a protective role in sarcopenia [21–23]. Thus, it is possible that galectin-1 is altered by PA as a response to normalized immunometabolism.

The aim of this study was to investigate the association between CRF and circulating galectin-1 levels and the interconnection with body fatness in middle-aged Swedish men and women.

## Methods

### Study overview

This is a cross-sectional study of data collected in the Swedish CArdioPulmonary bioImage Study (SCAPIS) pilot. CRF was used as an indirect measure of the PA level and BMI as an indirect measure of body fatness. The association between CRF and Galectin-1 was analyzed in linear regression modeling, including a hypothetical indirect association through BMI (mediation analysis). In addition, categories of CRF and BMI were created and compared. Age and sex were controlled for in all analyses.

### Study sample

The SCAPIS is a multicenter cohort of randomly selected men and women aged 50–64 years from six regions in Sweden [24]. A pilot study was performed in 2012 as part of SCAPIS. It consisted of 1111 individuals recruited from a random sample of 2243 individuals of the same age range from the population register of the Gothenburg area. The sample was stratified for socioeconomic status to consider its association with lifestyle and risk for cardiopulmonary disease. No exclusion criteria were applied except inability to understand written and spoken Swedish for informed consent. The age range was selected to target a period were significant changes in lifestyle and risk of cardiopulmonary disease occur. An invitation was sent by mail. The recruitment period was from 14 February 2012 to 18 December 2012. Ethical approval was obtained from the Ethical Review Board in Umeå (2010-228-31M). All participants gave written informed consent. The initial sample recruited consisted of 1107 individuals (50.0% females, aged 57.7 (4.4) years). Due to missing data on galectin-1, CRF and anthropometrics, the final sample analyzed was 774 individuals (49.6% females, aged 57.3 (4.4) years). There was an even distribution across the age range in both men and women.

### Measurements

A 100 mL venous blood sample was collected after an overnight fast for immediate analysis and stored in a biobank for later analysis [24]. Serum galectin-1 concentration (ng·ml^-1^) was determined using the Human Galectin-1 Quantikine ELISA Kit (R&D Systems, Minneapolis, MN, USA) following the manufacturer´s instructions [9]. Intra- and inter-assay coefficients of variation were 7.1% and 9.5%, respectively.

Cardiorespiratory fitness (VO_2_max, ml·kg^-1^·min^-1^) was estimated from the two-point Ekblom-Bak submaximal exercise test on a cycle ergometer (Monark, model 828E, Varberg, Sweden). The exercise test is performed on one lower standard load and one higher individually adapted load corresponding to at least moderate intensity. Heart rate is monitored during the test (Polar Electro, Kempele, Finland). The two heart rates and workloads are used to determine the slope (ΔHR/ΔPO), which is included in sex-separated algorithms together with age to estimate CRF. The algorithms has been validated towards measured VO_2_max in the age range corresponding to SCAPIS [25]. In this age range, there was a non-significant mean (95% confidence interval) difference of 0.08 (-0.11 to 0.27) L·min^-1^, a coefficient of variation of 11.8% and explained variance of 84%. The test is therefore considered to provide a feasible and valid estimation of VO_2_max. Body weight (kg) and height (m) was measured according to standard clinical procedures [24]. BMI was calculated as kg·m^-2^.

### Statistical analyses

Linear regression analysis was performed with CRF as predictor and galectin-1 as outcome, controlled for age and sex (Model 1). In a second step, BMI was added to the model (Model 2). An additional linear regression analysis was performed with CRF as predictor and BMI as outcome, controlled for age and sex (Model 3). The hypothetical indirect association between CRF and galectin-1 through BMI was calculated as the product of the regression coefficient of BMI in Model 2 and the regression coefficient of CRF in Model 3 (mediation analysis) [26]. The remaining direct association between CRF and galectin-1 was calculated as the regression coefficient in Model 1 minus the regression coefficient of the indirect association.

The sample was divided into two groups of equal size by CRF (Low, High) and by BMI (Low, High), sex separated. Four groups of CRF-BMI were thereafter created: Low-Low, Low-High, High-Low and High-High. One-way ANOVA with Bonferroni correction for multiple tests was used to investigate group differences in galectin-1. Statistical significance was set at p<0.05. All statistical analyses were performed in IBM SPSS Statistics version 29 (IBM Corporation, Armonk, NY, USA).

## Results

**Fig 1:**
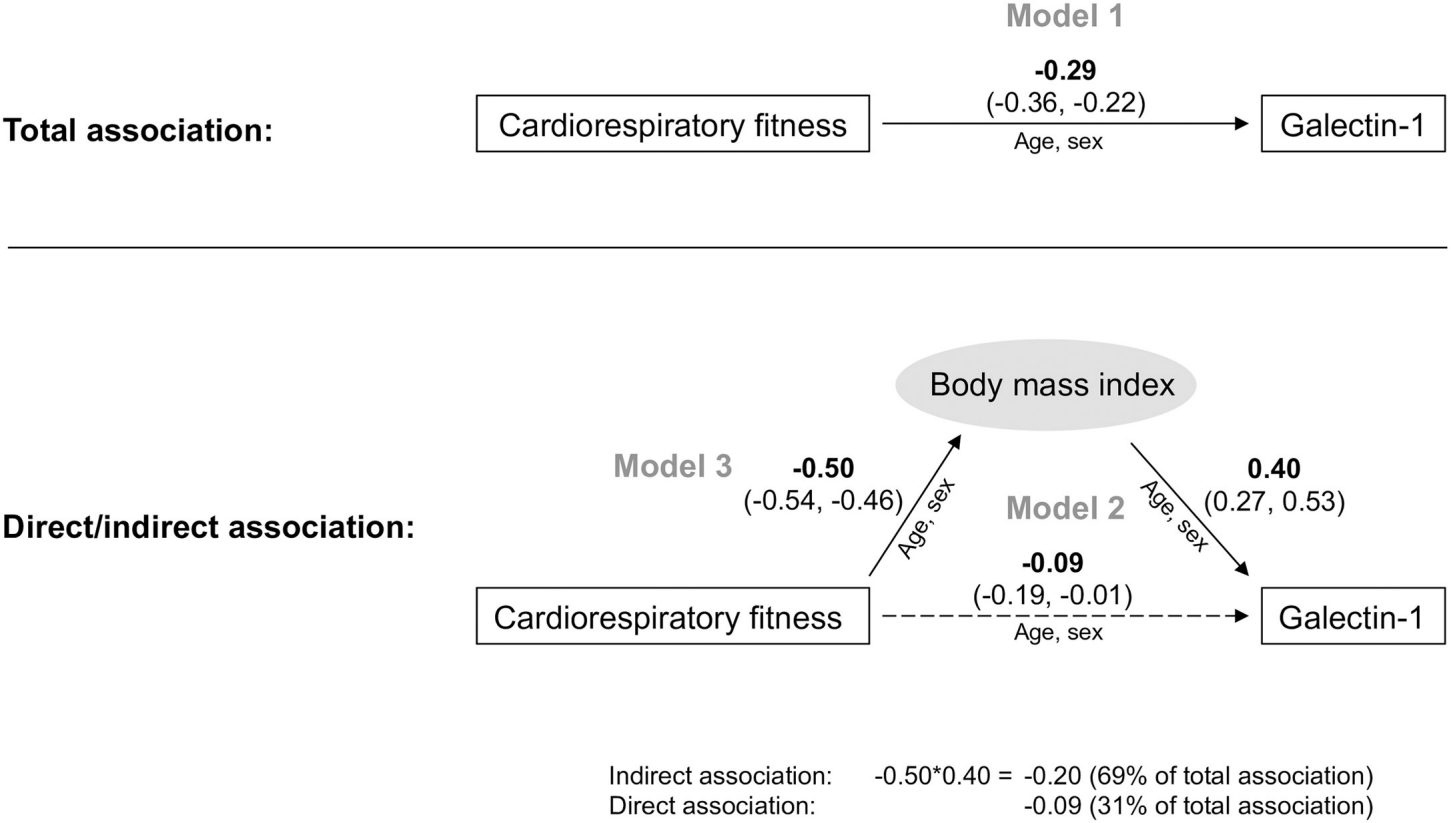
Regression analyses of cardiorespiratory fitness and galectin-1 and the indirect association through body mass index. Regression coefficient (95% confidence interval). Regression models controlled for age and sex. Model 1 shows the overall association between cardiorespiratory fitness and galectin-1. Model 2 adds body mass index. Model 3 is the association between cardiorespiratory fitness and body mass index. The indirect association through body mass index is calculated as the product of the regression coefficient from Model 2 and Model 3 (0.40 x -0.50 = -0.20).

Table 1 presents the characteristics of the entire study sample as well as the four CRF-BMI groups. The outcome of the linear regression modeling is displayed in Fig 1. There was a statistically significant (p<0.001) overall negative association between CRF and galectin-1 (Model 1). When BMI was added to the model the association of CRF was attenuated (p = 0.07, Model 2). The positive association between BMI and galectin-1 remained statistically significant (<0.001). Further, there was a statistically significant negative association between CRF and BMI (p<0.001, Model 3). The hypothetical indirect association (mediation) between CRF and galectin-1 through BMI was determined as the product of the regression coefficient for CRF in Model 3 and the regression coefficient for BMI in Model 2, i.e. -0.50 * 0.40 = -0.20. The indirect association constituted 69% of the overall association (0.20 / 0.29 = 0.69), while the remaining direct association constituted 31%.

**Table 1:** Characteristics of study sample and groups of cardiorespiratory fitness and body mass index.

Variable	Total sample N = 774	High CRF Low BMI N = 282	Low CRF Low BMI N = 105	High CRF High BMI N = 105	Low CRF High BMI N = 282
Age, years	57.2 (4.4)	56.7 (4.2)	59.4 (4.3)	55.3 (3.5)	57.8 (4.4)
Sex, % female	49.6	52.1	42.9	42.9	52.1
Galectin-1, ng·ml^-1^	22.6 (6.4)	20.6 (3.7)	21.9 (4.9)	22.15 (4.0)	25.0 (8.6)
CRF, ml·kg^-1^	32.0 (6.5)	37.3 (4.4)	28.7 (4.1)	35.1 (3.8)	26.6 (4.3)
BMI, kg·m^-2^	27.0 (4.4)	23.5 (2.0)	24.6 (1.6)	28.3 (1.8)	30.9 (1.8)

Mean (sd) or percent. BMI, Body Mass Index; CRF, cardiorespiratory fitness.

**Fig 2:**
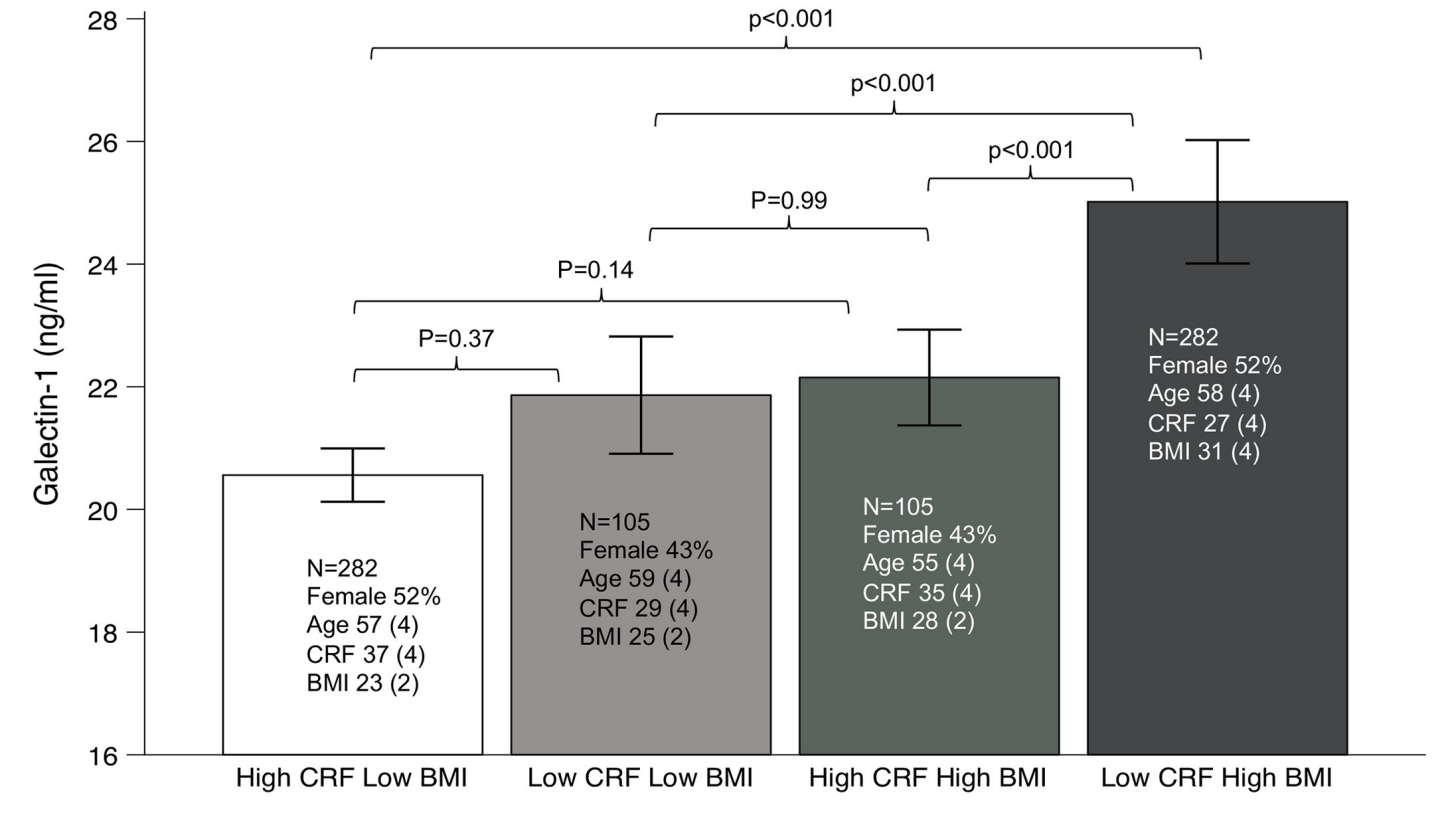
Groups by cardiorespiratory fitness (CRF) and body mass index (BMI), and the difference in serum galectin-1 (mean, 95% confidence interval).

Fig 2 presents group comparisons of galectin-1. The High CRF-Low BMI group had the lowest level of galecin-1, while the Low CRF-High BMI had the highest level. These groups were similar in age and sex distribution. The levels of galectin-1 for the other two groups were intermediate. The galectin-1 level in the High CRF-Low BMI was significantly different from the other three groups, while this was not the case between these three groups.

## Discussion

This cross-sectional study of middle-aged individuals in the Gothenburg area, showed that higher CRF (an indirect measure of being more physically active) was associated with lower circulating levels of galectin-1. It further showed that the interconnection between CRF and BMI explained a significant part of this association, and that the combination higher CRF and lower BMI contributed to the lowest galectin-1 levels.

High serum levels of galectin-1 observed in obesity, is a risk factor for developing T2D over time and is associated with poor outcome in several malignant cancers including prostate cancer and ovarian cancer [7, 9, 27–29]. It has previously been reported that the risk of incident T2D was proportional to the increase in circulating galectin-1 levels [7]. This indicates that it may be better from a clinical standpoint to have lower levels of circulating galectin-1. Currently, galectin-1 inhibitors are being developed in clinical trials to allow for the suppression of galectin-1 when motivated, and an oral galectin-1 inhibitor was recently discovered [30, 31]. Here, it appears that lower serum galectin-1 levels may be associated with a physically active lifestyle increasing CRF level. This would provide an alternative route for galectin-1 modulation *in vivo*. It is previously known that PA can reduce the risk of both T2D and different cancer types [18, 32]. It is possible that a suppression of galectin-1 could constitute a part of the protective effects of PA on these outcomes. The inverse association between serum galectin-1 and CRF has not been studied previously. However, a recent population-based study reported that the level of another galectin, galectin-3, was lower in individuals with a higher CRF, which is similar to our observation on galectin-1 [33]. While galectins all have unique roles in specific cells, it is also known that they present overlapping functionality in certain contexts [34].

Our observation of lower circulating galectin-1 in individuals with higher CRF is likely explained by several independent mechanisms. Based on our linear regression models, 2/3 of the association between galectin-1 and CRF are mediated through BMI, likely through mechanisms related to energy balance. Galectin-1 has a proposed role in the adipose tissue through lipid uptake and adipogenesis [35, 36]. In addition, 1/3 of the association between CRF and galectin-1 is direct or mediated by other factors than BMI. It is possible that this association is related to known effects by galectin-1 in skeletal muscle. High galectin-1 is found in sarcopenia and as a response to muscular degeneration and inflammation [37]. PA may suppress galectin-1 due to healthier muscular tissue. Alternatively, exercise reduces low grade systemic inflammation. It is possible that a reduced inflammation also results in lower levels of galectin-1 as seen in other studies on galectin-1 and different inflammatory diseases [34, 38, 39]. While there is substantial evidence on how galectin-1 modulates inflammation, studies are needed to further elucidate the direct effects of galectin-1 in healthy muscle physiology [40]. High intensity training increases CRF, which in turn is causally associated with lower risk of T2D in genetic studies [41–43]. As galectin-1 is a proposed biomarker in T2D, future studies should examine the potential biomarkers capabilities of serum galectin-1 in exercise interventions in patients with T2D.

Some limitations of this study need to be mentioned. The study was conducted in middle-aged individuals, and most participants were of European ancestry. It is possible that studies on younger individuals or individuals from other parts of the world may find different results. The study sample is relatively homogenous, reducing group differences or strength of associations between variables. Residual confounding most likely explains why our statistical models do not fully explain the observed serum galectin-1 level. Serum galectin-1 is known to correlate with several soluble immune markers and T-cell subpopulations [44], however, not measured in this cohort. Furthermore, this is a cross-sectional study, preventing any causal conclusions. Intervention and mechanistic studies on PA may evaluate changes in serum galectin-1 and determine causality of PA on serum galectin-1. A more physically active lifestyle increasing CRF may reduce body fatness, as well as lower body fatness may facilitate a physically active lifestyle increasing CRF.

In summary, this study provides new insights into the role of galectin-1 in human physiology and suggests that galectin-1 could be a marker of the beneficial health effects of PA. Our results add to previous knowledge about the role of galectin-1 in energy homeostasis through caloric intake [8, 44], suggesting that it is also regulated by PA. This study suggests that it may be possible to modulate serum galectin-1 levels through PA affecting body fatness, with potential benefits to human health. Still, the health implications remain to be confirmed in intervention studies, connecting improvement in PA/CRF and body fatness with reduction in galactin-1 levels.
